# Olfactory Groove Meningioma Presenting With Visual Decline and Gait Disturbance: A Case Report

**DOI:** 10.7759/cureus.103165

**Published:** 2026-02-07

**Authors:** Elizabeth Blanco Espinosa, Idania Cruzata Matos, Lauren Lopez, Hiran A Morales, Veronica A Somoza Chousa

**Affiliations:** 1 General Practice, CEDA Orthopedic Group, Miami, USA; 2 General Surgery, Hospital Universitario Arnaldo Milian Castro, Santa Clara, CUB; 3 General Medicine, HCA Healthcare, Las Vegas, USA; 4 Neurology, Neurology of Central Florida, Orlando, USA; 5 Internal Medicine, Universidad de Ciencias Médicas de Ciego de Ávila, Ciego de Ávila, CUB; 6 General Medicine, Universidad de Ciencias Médicas Santiago de Cuba, Santiago de Cuba, CUB

**Keywords:** anosmia, anterior cranial fossa, bifrontal craniotomy, frontal lobe syndrome, olfactory groove meningioma, visual loss

## Abstract

Olfactory groove meningiomas (OGMs) are typically indolent neoplasms arising from the anterior skull base and frequently attain a considerable size prior to detection because early symptoms are often vague and nonspecific. Common clinical features include personality or behavioral alterations, diminished or absent olfaction, and gradually worsening visual impairment, which are often incorrectly attributed to psychiatric conditions or neurodegenerative processes, leading to delays in diagnosis.

We report the case of a 55-year-old female who presented with a seven-month history of progressive bilateral visual decline, unilateral headaches, anosmia, and gait instability. Computed tomography (CT) revealed a well-defined, isodense frontobasal mass. Computed tomography angiography (CTA) confirmed a hypervascular lesion compatible with OGM. The patient underwent bifrontal craniotomy with a bitemporal incision, with preservation of the temporalis fascia and drilling of the tumor base to minimize recurrence. Postoperatively, the patient showed partial visual improvement and significant recovery of orientation and gait. Histopathology confirmed a WHO Grade I meningioma.

This case underscores the diagnostic value of subtle frontal signs - anosmia, disorientation, and gait disturbance - in middle-aged or older adults with cognitive or visual complaints. Timely radiological assessment enables early identification of OGMs before irreversible deficits occur. The bifrontal approach with basal bone drilling remains a safe and effective strategy for achieving gross total resection while minimizing recurrence risk.

## Introduction

Olfactory groove meningiomas (OGMs) account for approximately 10%-15% of all intracranial meningiomas and arise from the dura of the crista galli and the floor of the anterior cranial fossa, representing a well-defined subset of anterior skull base meningiomas [1,2]. They are more frequently observed in middle-aged women and typically grow slowly, allowing these tumors to reach large sizes before producing evident symptoms [3]. Because of the spacious anatomy of the anterior cranial fossa, OGMs may remain clinically silent for long periods, often leading to delayed diagnosis.

Clinically, OGMs frequently present with subtle or nonspecific manifestations, such as behavioral changes, personality alterations, and olfactory dysfunction - features that may mimic psychiatric or neurodegenerative disorders. Compression of the frontal lobes and olfactory apparatus results in anosmia or hyposmia, while posterior extension can compromise the optic apparatus, producing bitemporal visual field deficits. In some cases, mass effect on medial frontal motor pathways may also lead to gait disturbances, such as frontal gait apraxia, highlighting the importance of comprehensive neurological assessment [1,3].

Neuroimaging typically reveals a midline frontobasal mass with perilesional edema and, in many cases, hyperostosis or bony invasion of the anterior skull base [4]. Surgical resection remains the primary treatment for OGMs, but it poses several challenges, including preservation of olfactory function, minimizing frontal lobe manipulation, and addressing bone invasion to reduce recurrence [5]. Multiple surgical approaches have been described, including unilateral or bilateral transcranial, endoscopic endonasal, and transorbital approaches, each with distinct advantages and limitations. This case uniquely illustrates the combination of severe visual loss, prominent neuropsychiatric features, and imaging-based surgical planning in a setting where only computed tomography (CT) imaging was available [6,7].

In this context, we present a 55-year-old female with a large OGM, with progressive visual loss, olfactory disturbance, and gait impairment - an uncommon clinical combination that underscores the need for early recognition of subtle frontal and anterior fossa symptoms to prevent irreversible deficits. The patient underwent a bifrontal craniotomy with basal bone drilling, consistent with contemporary surgical strategies for anterior skull base meningiomas [3,4]. This case provides additional evidence from a Latin American neurosurgical setting, demonstrating the importance of thorough clinical evaluation and CT-based surgical planning when magnetic resonance imaging (MRI) or advanced imaging may not be immediately available.

## Case presentation

History and clinical findings

A 55-year-old woman with a history of migraines was brought to the neurology clinic by her daughter due to progressive bilateral visual decline over the preceding eight months. The patient reported increasing difficulty recognizing faces at short distances, impaired reading ability, and frequent collisions with surrounding objects while walking. She also described unilateral frontal headaches, worse in the mornings, accompanied by a sensation of retro-orbital pressure. Family members noted progressive behavioral changes over the past six months, including apathy, reduced social interaction, and episodes of disinhibition. The patient additionally endorsed progressive anosmia over the past year, which she had initially attributed to allergic rhinitis. Over the preceding three months, she also experienced intermittent nausea, mild memory impairment, reduced attention span, and subjective cognitive slowing. There was no history of seizures, head trauma, visual aura, fever, weight loss, or other systemic symptoms. On neurological examination, the patient was awake and cooperative, but exhibited a flat affect and bradyphrenia. Clinical cognitive assessment revealed mild impairment, predominantly affecting attention, executive functioning, and processing speed, while orientation and language abilities were relatively preserved. A frontal release sign was present, evidenced by a positive grasp reflex. Cranial nerve examination revealed bilateral optic disc pallor on funduscopic examination, decreased visual acuity (20/200 bilaterally), and bitemporal visual field deficits, more pronounced in the inferior quadrants on confrontation testing. Pupillary light reflexes were sluggish but symmetric. Extraocular movements were intact. Olfactory function was assessed using non-irritant odor identification testing (coffee and soap), confirming bilateral anosmia.

Motor and sensory examinations were normal, with preserved limb strength and reflexes. Specifically, the patient exhibited a hesitant, wide-based gait with difficulty initiating steps and reduced arm swing, without dysmetria, intention tremor, or truncal instability, supporting a diagnosis of frontal gait apraxia rather than true ataxia. Gait instability was objectively quantified using the Tinetti Performance-Oriented Mobility Assessment, with a score of 23/28, consistent with mild gait disturbance [8]. This scale has been validated for the assessment of gait and balance disorders associated with frontal lobe dysfunction, and provides a reproducible measure of functional impairment.

Given the constellation of progressive bilateral visual loss, anosmia, frontal lobe signs, behavioral changes, and gait disturbance, a structural lesion involving the anterior cranial fossa and frontobasal region was strongly suspected. The differential diagnosis included intracranial mass lesions such as OGM, pituitary macroadenoma with suprasellar extension, and frontal lobe glioma. Neurodegenerative disorders, including frontotemporal dementia, were considered less likely due to the presence of objective visual field defects, cranial nerve involvement, and radiologic features suggestive of mass effect with multidirectional displacement of adjacent structures, rather than isolated superior displacement. Chronic inflammatory or granulomatous processes were considered less probable in the absence of systemic manifestations.

Imaging findings

**Figure 1:**
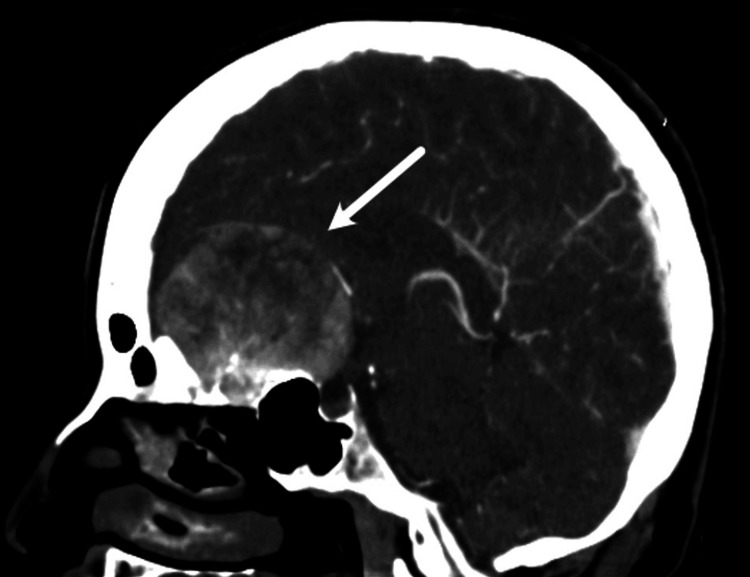
Non-contrast sagittal CT of the brain demonstrates a well-defined, hyperdense suprasellar mass, with significant mass effect on adjacent structures (arrow). CT, computed tomography

Non-contrast CT of the brain demonstrates a large, well-circumscribed extra-axial mass located in the anterior cranial fossa, centered along the olfactory groove. The lesion appears isodense to mildly hyperdense relative to the adjacent brain parenchyma. The mass exerts a significant multidirectional mass effect, including bilateral posterior displacement and compression of the frontal lobes, inferior displacement of the optic nerves and optic chiasm, and compression of the anterior horns of the lateral ventricles without frank obstructive hydrocephalus (Figure 1). 

**Figure 2:**
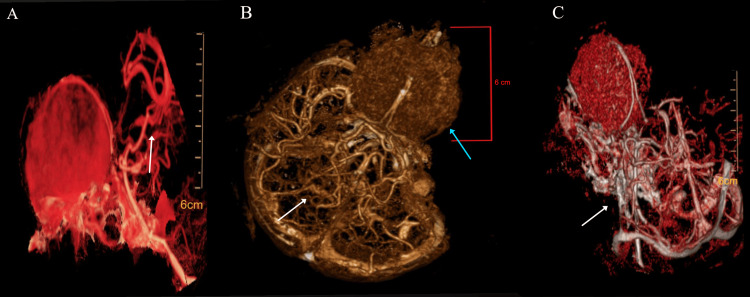
CTA confirmed a vascularized lesion in the olfactory groove region suggestive of a meningioma. (A) Volume-rendered CTA demonstrates a hypervascular, extra-axial lesion located in the olfactory groove region, with arterial supply predominantly arising from the bilateral anterior ethmoidal arteries (branches of the ophthalmic arteries), as indicated by the white arrow. Additional arterial contribution from the ACA, including frontopolar and orbitofrontal branches, is also visualized. These vascular features are suggestive of a meningioma. (B) Three-dimensional volumetric reconstruction highlights the solid tumor component (blue arrow), measuring approximately 6 cm, and the dense cortical vascular network (white arrow), corresponding to feeding branches from the anterior cerebral circulation and ethmoidal arteries. (C) Combined volume-rendered CTA shows prominent intratumoral enhancement, with clearly identifiable arterial feeders and early draining veins (white arrow). Venous outflow is observed through superficial frontal cortical veins, with drainage toward the superior sagittal sinus, underscoring the lesion’s hypervascular nature and its relevance for surgical planning. CTA, computed tomography angiography; ACA, anterior cerebral artery

Computed tomography angiography (CTA) revealed a hypervascular lesion with arterial supply predominantly from the bilateral anterior ethmoidal arteries (branches of the ophthalmic arteries), and additional contributions from the anterior cerebral artery (frontopolar and orbitofrontal branches). Venous drainage occurred via superficial frontal cortical veins toward the superior sagittal sinus, findings relevant for surgical planning. According to the Al-Mefty vascular classification for anterior skull base meningiomas, the tumor demonstrated a predominantly ethmoidal-ophthalmic arterial supply, consistent with olfactory groove origin and supporting the feasibility of early devascularization during bifrontal exposure [9]. CTA was considered sufficient for vascular assessment, consistent with current standards (Figures 2A-2C). 

Surgical procedure

**Figure 3:**
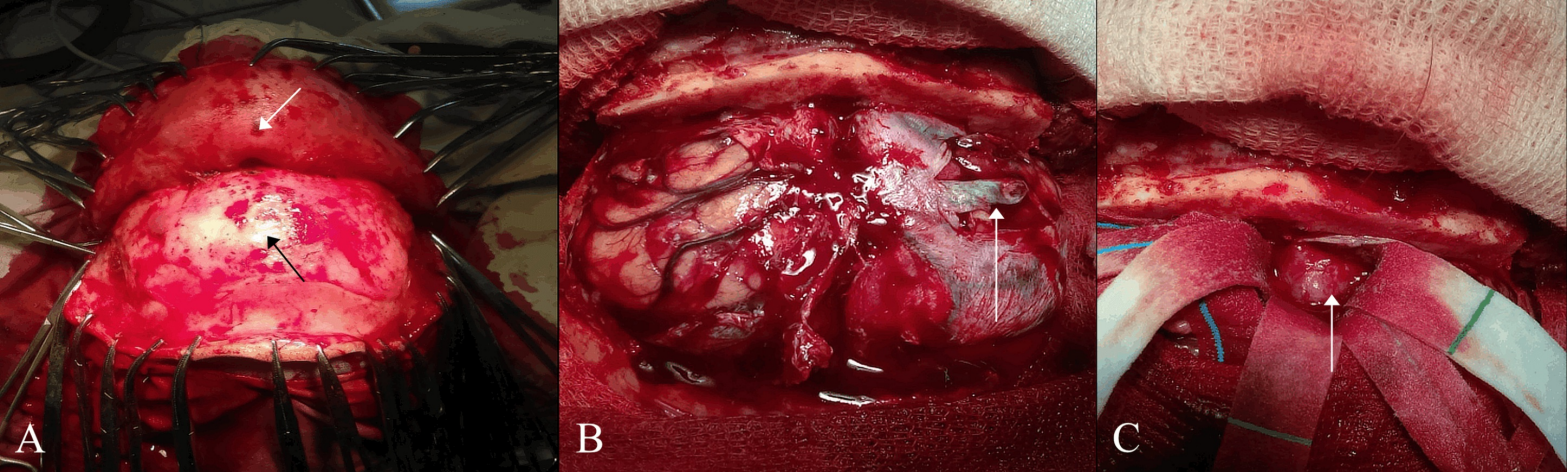
Intraoperative views of the surgical procedure. (A) Bifrontal craniotomy with temporal incision. The white arrow indicates the pericranial flap displaced anteriorly, and the black arrow shows the frontal bone. (B) Intraoperative view after dural opening. The white arrow shows the frontal lobe. (C) Surgical field, with the white arrow showing the tumor.

The patient was positioned supine, with the head slightly extended and secured in a Mayfield clamp. A standard bicoronal incision was performed, and the scalp and temporalis muscles were elevated in a subgaleal plane to expose the frontal bone. A bifrontal craniotomy was performed, and the bone flap was removed. Prior to dural opening, the anterior portion of the superior sagittal sinus was carefully identified, protected, and preserved throughout the procedure. The dura was then opened anteriorly in a curvilinear fashion (Figures 3A-3C).

Microsurgical tumor resection was performed under operative microscopy. Initial internal debulking of the tumor was carried out, followed by progressive circumferential dissection from the frontal lobes and anterior skull base. The optic nerves, optic chiasm, and anterior cerebral arteries were identified and dissected last, once adequate tumor decompression had been achieved, minimizing traction on critical neurovascular structures.

**Figure 4:**
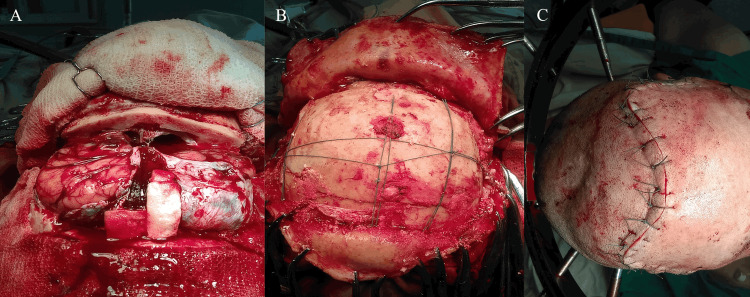
Intraoperative stages of cranial reconstruction. (A) Surgical field after completion of tumor resection, showing the exposed frontal lobes prior to reconstruction. (B) Replacement of the bifrontal bone flap and fixation using cranial sutures, restoring the cranial vault. (C) Final appearance after scalp closure, demonstrating satisfactory wound approximation and cranial contour restoration.

Hyperostotic bone involving the cribriform plate and frontobasal region was drilled down to normal cortical bone to ensure complete resection. Gross-total tumor removal, corresponding to Simpson grade I resection, was achieved. The dura was closed primarily with pericranial graft augmentation, and the bone flap was secured using sutures in a crisscross fashion, without the use of rigid fixation devices such as plates or screws. The wound was closed in anatomical layers. The patient tolerated the procedure well and was transferred to recovery uneventfully. No intraoperative complications were reported (Figures 4A-4C).

Histopathology confirmed a WHO Grade I meningioma. Postoperatively, the patient experienced gradual improvement in her headaches and executive functioning. However, anosmia persisted, and visual recovery remained limited. To objectively assess visual recovery, the patient underwent formal visual field testing (perimetry), which demonstrated partial improvement in the peripheral visual fields.

Ethical approval

Approval from the ethics committee was not necessary for this individual case report, following the guidelines of the local institution. Written informed consent was obtained from the patient for the publication of de-identified clinical information and associated images. All efforts were made to preserve the anonymity and privacy of the patient, and no identifying personal data has been included in this manuscript. 

## Discussion

GMs represent approximately 10% of intracranial meningiomas, with a mean age of onset around 54 years and a strong female predominance, consistent with our 55-year-old female patient [10].

A characteristic symptom constellation includes gradual loss of smell, progressive visual impairment, and changes in behavior or personality. These clinical features reflect early compromise of the olfactory pathways, mass effect on the optic system, and involvement of frontal lobe circuits. Headache is frequently reported, occurring in approximately 31%-86% of patients, while olfactory deficits are described in 57%-78%, and behavioral or personality changes in 48%-72%. Additional clinical features may include varying degrees of visual dysfunction (24%-61%), epileptic seizures (17%-35%), or manifestations of elevated intracranial pressure, reported in up to 50.8% of cases. Notably, 3%-12% of OGMs are incidentally identified during neuroimaging obtained for unrelated clinical indications [10]. The present case illustrates this characteristic triad but also demonstrates several atypical and clinically significant features that add relevance to the differential diagnosis and management of OGMs.

In large published series, the symptom pattern of our patient aligns with previously reported clinical trends but also demonstrates notable differences in severity. Spektor et al., in a cohort of 80 patients, reported visual impairment in 47.5% and personality or behavioral changes in 40% of cases [11]; in contrast, our patient exhibited more profound bilateral visual loss (20/200) and marked behavioral dysfunction. Similarly, Zhang et al. reported vision loss in 51% and behavioral changes in 44% among 79 patients [12], yet few of their patients exhibited the combination of severe visual decline with prominent apathy and disinhibition seen in our case. Niklassen et al. highlighted that anosmia is commonly documented, but neuropsychiatric symptoms are often present, yet underemphasized, in case reports [13]. Additionally, Ikhuoriah et al. described visual impairment ranging from 24% to 61% and personality change in nearly half of patients [10], underscoring the variability of clinical presentation. Compared with these series, our case illustrates a more pronounced constellation of sensory and behavioral symptoms, emphasizing the heterogeneous nature of OGMs [14].

Our patient also presented with gait disturbance, which can be attributed to mass effect from the olfactory groove lesion extending posteriorly toward the frontal lobes and adjacent medial motor pathways, potentially causing frontal gait apraxia. Formal neurological assessment revealed a wide-based, hesitant gait with difficulty initiating steps, consistent with frontal gait dysfunction rather than cerebellar ataxia. From a pathophysiological standpoint, compression of the medial frontal cortex, supplementary motor area, and frontopontine connections disrupts higher-order gait initiation and motor planning, leading to the characteristic “magnetic” or hesitant gait observed in frontal lobe syndromes. Such gait abnormalities are increasingly recognized in large anterior skull base tumors and may precede overt motor weakness. MISME (Multiple Inherited Schwannomas, Meningiomas, and Ependymomas) syndrome was considered in the differential but was ruled out based on clinical, imaging, and laboratory findings [15].

First, the progressive bilateral visual decline, combined with pronounced bitemporal hemianopic deficits, underscored the substantial mass effect exerted by the lesion on the optic chiasm. Although OGMs may compromise vision when large, this patient presented with severe bilateral visual impairment (20/200) at initial evaluation, a less frequent presentation typically correlating with advanced tumor size or delayed detection. The coexistence of anosmia, frontal release signs, executive dysfunction, and gait disturbance strengthened the suspicion of an anterior skull base tumor, even before neuroimaging.

Alterations in olfactory function, ranging from partial smell reduction to complete loss, commonly appear early in OGMs and have been reported in approximately 58.5%-71.7% of affected patients. However, isolated olfactory complaints are the initial clinical manifestation in only a minority of cases (6.4%-15%). Rather than occurring alone, olfactory deficits are frequently accompanied by visual abnormalities, headache syndromes, behavioral changes, and, as in this case, gait disturbance. In clinical series, headache - often followed by neurobehavioral alterations - has been identified as the most frequent initial presentation. Visual dysfunction occurs in roughly one-quarter to over half of patients with OGMs (24%-61%) [6,10,11,15].

A particularly noteworthy and distinctive feature of this case is the degree of behavioral and neurocognitive impairment, including apathy, social withdrawal, and episodes of disinhibition. These symptoms are commonly seen in frontal lobe syndromes but are underreported in OGM literature, which occasionally focuses more on visual outcomes. The inclusion of overt frontobasal neuropsychiatric manifestations and frontal gait abnormalities highlights the need for clinicians to consider OGMs in the differential diagnosis of progressive behavioral changes and motor dysfunction in middle-aged or older adults, especially when accompanied by visual or olfactory deficits.

MRI is considered the preferred technique for evaluating OGMs and, in combination with magnetic resonance angiography (MRA), provides detailed visualization of the tumor’s spatial relationship with critical neurovascular structures, including the optic nerves, optic chiasm, and the anterior cerebral and anterior communicating arteries. Given its noninvasive nature and diagnostic accuracy, MRA has largely supplanted traditional catheter-based angiography, while still allowing assessment of tumor vascular supply and dominant feeding vessels [10]. CTA is now considered sufficient and is the gold standard for preoperative vascular evaluation, replacing catheter studies in most cases. However, digital subtraction angiography (DSA) remains the formal gold standard for detailed vascular characterization, particularly when precise delineation of tumor feeders, venous drainage, or preoperative embolization is contemplated. In many contemporary settings, CTA offers a reliable, non-invasive alternative that balances diagnostic accuracy with procedural safety, especially in resource-limited environments.

CT imaging is highly sensitive (>85%) for detecting hyperostosis, bone invasion, calcifications, and significant mass effect - features strongly suggestive of an OGM. MRI achieves >90% sensitivity for delineating dural tails, optic pathway compression, and peritumoral edema. In resource-limited settings or when MRI is delayed, CT and CTA provide reliable diagnostic information and were essential in this case for surgical planning and identification of tumor vascularization.

From a radiological perspective, this case illustrates classic features of OGMs: a large, well-defined extra-axial mass with extensive dural contact, hyperdense signal on non-contrast CT, uniform enhancement after contrast, perilesional vasogenic edema, and slight hyperostosis of the cribriform plate [16]. Notably, the CTA revealed a prominent vascular pattern, highlighting the lesion’s high vascularity, which is crucial for surgical planning [17]. 

Several alternative surgical approaches have been described for OGMs, each offering specific advantages depending on tumor size, extension, and surgeon experience. The unilateral frontolateral approach provides shorter operative times and less brain retraction, but offers limited exposure for large midline tumors. The supraorbital “keyhole” eyebrow approach minimizes soft tissue disruption and is suitable for small- to medium-sized OGMs, though it restricts visualization of the contralateral olfactory tract and anterior falx. The endoscopic endonasal approach (EEA) is increasingly used for midline tumors confined to the anterior cranial fossa floor, allowing early devascularization, but carrying higher risks of cerebrospinal fluid (CSF) leak and limited lateral reach. The transorbital approach provides anterolateral access with minimal brain manipulation, but is typically reserved for small lesions. In our patient, the bifrontal approach was required due to the large tumor volume, bilateral optic nerve compression, superior extension, and hyperostotic bone requiring extensive drilling, making minimally invasive or unilateral routes inadequate for safe gross-total resection. 

In OGMs, hyperostosis of the cribriform plate and adjacent frontal bone is frequently caused by true bone invasion rather than a purely reactive change. Histopathological studies have shown that up to 60% of hyperostotic bone contains microscopic nests of meningioma cells [18]. Therefore, drilling or resection of the involved basal bone reduces the risk of recurrence by removing residual tumor cells located within the diploë, osteons, and dural attachment sites. This approach enables a Simpson grade I resection, which has significantly lower long-term recurrence rates when compared with Simpson grades II-III. The aggressive management of hyperostotic bone is particularly important in anterior skull base tumors, where the dural implantation may extend beyond the visible tumor margins. Consequently, drilling the cribriform plate and frontobasal bone during bifrontal craniotomy contributes directly to recurrence prevention. A notable aspect of this case is the distinction between the technique applied and currently preferred neurosurgical methods. Even though the intervention took place in a Latin American context, the meticulous application of surgical fundamentals ensured a high-quality procedure and an exemplary postoperative evolution. This reinforces the value of solid neurosurgical principles, regardless of geographic or resource-related differences. 

Postoperative improvement in headaches and executive functioning reflects successful decompression of frontal lobe structures, although the persistence of anosmia is common due to irreversible olfactory nerve damage. Comprehensive assessment of olfactory function is not routinely incorporated into the evaluation of patients with OGMs, and postoperative smell outcomes are inconsistently measured and documented. Surgical management is associated with a substantial likelihood of partial or complete impairment of olfaction, reported in rates as high as 89.7%. Nevertheless, because systematic olfactory testing is infrequently performed both before and after intervention, the actual prevalence of baseline dysfunction remains unclear, and postoperative deterioration of smell is likely underestimated in the literature.

Visual outcomes following OGM resection vary widely. Across published series, approximately 40%-60% of patients experience postoperative visual improvement, 20%-40% remain stable, and 10%-20% deteriorate. Predictors of favorable recovery include shorter duration of visual symptoms, absence of optic disc pallor, younger age, and complete resection without manipulation of the optic pathways. Poor prognostic indicators include severe preoperative visual loss, long-standing compression, and optic nerve thinning. In this case, partial recovery of peripheral fields aligns with typical outcomes for advanced preoperative visual impairment and some degree of optic nerve resilience, despite prolonged compression.

Another interesting aspect is that the patient’s initial symptoms were attributed to migraines and allergies, contributing to a delay in specialist evaluation. This highlights the diagnostic challenge of OGMs, which often mimic more benign or nonspecific conditions during early stages. Early recognition of the symptom constellation - especially progressive anosmia, frontal behavioral changes, and visual decline - could facilitate earlier imaging and potentially improve visual outcomes [19].

This report highlights the need for heightened clinical awareness of anterior cranial base pathology when visual impairment, olfactory dysfunction, and behavioral changes coexist. It further emphasizes the diagnostic value of CT and CTA, the importance of distinguishing neurological disease from primary psychiatric presentations, and the necessity of early neurosurgical management to reduce the risk of permanent visual deficits.

## Conclusions

OGMs represent a diagnostic challenge due to their indolent growth and often deceptive clinical manifestations. The combination of visual impairment, olfactory dysfunction, and mild cognitive or gait disturbances should raise suspicion for a frontobasal lesion, particularly in older adults. This case illustrates how early recognition of these subtle frontal signs can guide appropriate imaging, prevent diagnostic delay, and improve neurological outcomes. From a surgical standpoint, the bifrontal approach allows optimal exposure of the anterior cranial fossa, facilitating complete tumor removal and bone drilling of the implantation site - key factors in reducing recurrence. Moreover, reporting cases from Latin American neurosurgical centers contributes a valuable perspective to the global literature, emphasizing that successful outcomes can be achieved even in resource-variable settings, when meticulous microsurgical technique and multidisciplinary care are applied. 
